# Exploring Variations in Sleep Perception: Comparative Study of Chatbot Sleep Logs and Fitbit Sleep Data

**DOI:** 10.2196/49144

**Published:** 2023-11-21

**Authors:** Hyunchul Jang, Siwoo Lee, Yunhee Son, Sumin Seo, Younghwa Baek, Sujeong Mun, Hoseok Kim, Icktae Kim, Junho Kim

**Affiliations:** 1 KM Data Division Korea Institute of Oriental Medicine Daejeon Republic of Korea

**Keywords:** sleep, sleep time, chat, self-report, sleep log, sleep diary, wearables, Fitbit, patient-generated health data, PGHD

## Abstract

**Background:**

Patient-generated health data are important in the management of several diseases. Although there are limitations, information can be obtained using a wearable device and time-related information such as exercise time or sleep time can also be obtained. Fitbits can be used to acquire sleep onset, sleep offset, total sleep time (TST), and wakefulness after sleep onset (WASO) data, although there are limitations regarding the depth of sleep and satisfaction; therefore, the patient’s subjective response is still important information that cannot be replaced by wearable devices.

**Objective:**

To effectively use patient-generated health data related to time such as sleep, it is first necessary to understand the characteristics of the time response recorded by the user. Therefore, the aim of this study was to analyze the characteristics of individuals’ time perception in comparison with wearable data.

**Methods:**

Sleep data were acquired for 2 weeks using a Fitbit. Participants’ sleep records were collected daily through chatbot conversations while wearing the Fitbit, and the two sets of data were statistically compared.

**Results:**

In total, 736 people aged 30-59 years were recruited for this study, and the sleep data of 543 people who wore a Fitbit and responded to the chatbot for more than 7 days on the same day were analyzed. Research participants tended to respond to sleep-related times on the hour or in 30-minute increments, and each participant responded within the range of 60-90 minutes from the value measured by the Fitbit. On average for all participants, the chat responses and the Fitbit data were similar within a difference of approximately 15 minutes. Regarding sleep onset, the participant response was 8 minutes and 39 seconds (SD 58 minutes) later than that of the Fitbit data, whereas with respect to sleep offset, the response was 5 minutes and 38 seconds (SD 57 minutes) earlier. The participants’ actual sleep time (AST) indicated in the chat was similar to that obtained by subtracting the WASO from the TST measured by the Fitbit. The AST was 13 minutes and 39 seconds (SD 87 minutes) longer than the time WASO was subtracted from the Fitbit TST. On days when the participants reported good sleep, they responded 19 (SD 90) minutes longer on the AST than the Fitbit data. However, for each sleep event, the probability that the participant’s AST was within ±30 and ±60 minutes of the Fitbit TST-WASO was 50.7% and 74.3%, respectively.

**Conclusions:**

The chatbot sleep response and Fitbit measured time were similar on average and the study participants had a slight tendency to perceive a relatively long sleep time if the quality of sleep was self-reported as good. However, on a participant-by-participant basis, it was difficult to predict participants’ sleep duration responses with Fitbit data. Individual variations in sleep time perception significantly affect patient responses related to sleep, revealing the limitations of objective measures obtained through wearable devices.

## Introduction

Patient-generated health data play an important role in the management of many diseases. Various types of health-related information can be obtained from patients, ranging from subjective feelings or pain to objectively measurable steps and sitting times. Although some information remains unobtainable and there are various restrictions, it is possible to obtain diverse types of information from patients using wearable devices. In relation to sleep, time-related information such as the time an individual falls asleep and time they wake up can be obtained, as well as sleep quality and sleep environment information [1,2].

The current standard for clinical sleep evaluation is polysomnography (PSG) [3,4]. However, because PSG is usually performed in a hospital, actigraphy is used as an alternative in outpatient environments [5-7]. Actigraphy is less accurate than PSG, but is generally considered to be more accurate than sleep diaries. As PSG is performed in a sleep laboratory, many studies have used actigraphy to measure bedtime or wake-up time in everyday life and to study sleep-related diseases [8-13].

To track the state of sleep, an app installed on a smartphone or a sensor installed on a mattress or around it is used, although the accuracy of such devices is lower than that of a device worn directly [14,15]. In addition, the sleep state can be obtained through a sleep diary or questionnaire, which is less accurate but nevertheless useful in that the subjective sleep information of the user can be obtained [15]. As wearables still have limitations in assessing the depth or quality of sleep, there is a need to utilize the user’s perceived sleep, and user feedback is required until an objective diagnostic test technique can exclude the user’s subjective feelings. Each of these sleep measurement methods has advantages and disadvantages in terms of accuracy, convenience, and cost, and information can only be obtained through subjective methods. There is a need to use two or more methods together to create a synergistic effect between objective and subjective methods and to compensate for each of their disadvantages [15].

Commercially available wearables for actigraphy include various smart watches and fitness trackers. Although wearables are less reliable, they provide acceptable levels of sleep monitoring and are promising monitoring tools [16]. One representative wearable device is the fitness tracker Fitbit [17-25]. In addition to movement, Fitbits measure heart rate and other characteristics to provide sleep values [26-28]. Fitbit was reported to calculates total sleep time (TST) by 9 minutes more and sleep onset latency (SOL) by 4 minutes less compared with PSG, and a correlation between sleep onset, sleep offset, TST, and wakefulness after sleep onset (WASO) compared with PSG was reported [29]. Fitbit has been shown to be accurate to some extent for measuring sleep time, although there are limitations with sleep depth; however, there is no device that accurately measures sleep stages [30-34]. Therefore, a more personalized model is required to determine sleep stages or sleep quality using wearables [35].

Balancing user acceptance and monitoring performance is the biggest challenge in sleep-monitoring system research in terms of cost and efficiency [36]. A separate process such as charging and wearing may be required to wear a wearable device for a long period of time [37]. Wearables have several advantages, although they also have well-known disadvantages. There are many difficulties such as not wearing them, not wearing them properly, and the devices not accurately identifying the wearer. When using various devices, there are problems related to differences in operation methods or algorithms [38]; even when using a single device, the measurement process or results may change because of changes in firmware or algorithms. Therefore, further research on standardized performance evaluation systems for sleep-tracking technology is required [39].

In the United States, women sleep more on average than men [40]. Women also have better objective sleep quality, sleep duration, and sleep efficiency than men; however, they report poor sleep [41]. One study reported that subjective sleep quality was low in women [42]. In Australia, men stated that they think that their quality of sleep is better than that of women [43], and a report in China based on a Pittsburgh Sleep Quality Index (PSQI) survey suggested that women have worse quality of sleep than men [44]. Although many studies have addressed gender differences in sleep, few have addressed the differences between healthy men and women. In general, adult men and women require approximately 7 hours of sleep [45], and many websites do not distinguish the appropriate sleep times for adults by age. The difference in sleep time between the ages of 30 and 50 years is not large [46]. The role of BMI can vary depending on age, although it is considered that the higher the BMI, the shorter the sleep time and the lower the BMI, the longer the sleep time [45]. People with a high BMI of 30 kg/m^2^ have a slightly shorter than average sleep time [47]. People with obesity complain of insomnia or sleep disorders more often than those without obesity, and an association between obesity and increased daytime sleepiness or fatigue has been reported [48,49].

The difference between the amount of sleep measured by a Fitbit and how much sleep users feel they had is not well known. It is also not known how sleep time differs from day to day, other than rough information obtained through questionnaires. It is very important to understand how the perception of average sleep time, which reflects the quality of sleep for a certain period, differs from the daily recorded sleep time. The user’s recognition can be obtained through a sleep diary or survey, which also has limitations. Conversation apps offer a potential solution in this respect, which have been widely used recently and can be used to obtain periodic and immediate feedback. Therefore, it is necessary to compare the data obtained on the same day through chatbot conversations and Fitbit data to reveal more accurate user perception differences. However, to obtain daily information, wearables and daily user feedback are required, user convenience needs to be considered, and the user response must be minimized. Accordingly, the aim of this study was to analyze the characteristics of users’ time responses to sleep by comparing data obtained through Fitbit and chatbot conversations on the same day.

## Methods

### Recruitment

The Korean Medicine Daejeon Citizen Cohort study is being conducted over a 9-year period between 2017 and 2025, including 2000 adults living in Daejeon [50]. The cohort inclusion criteria are as follows: (1) men and women aged 30-55 years, (2) residents of Daejeon, and (3) individuals who provided informed consent. However, individuals are excluded if they (1) have been diagnosed with a malignant tumor or cardiovascular disease (myocardial infarction, angina, stroke/apoplexy); (2) are deemed to have difficulty following study instructions, such as having difficulty completing and understanding the questionnaire; or (3) determined by the researcher to be inappropriate to participate in this study. This study was conducted among the cohort participants who agreed to wear a Fitbit. For approximately 2 years, from October 10, 2020, to November 9, 2022, participants who agreed to participate in the PSQI survey, wear a Fitbit device, and have chatbot conversations were recruited, and sleep information was obtained. The participants were adults without special health problems who were in their 30s to 50s that agreed to wear a Fitbit and installed the Telegram-based chatbot app on their smartphone. The PSQI survey was conducted on the day of the hospital visit with those who wished to participate, and they were asked to wear a Fitbit device for approximately 2 weeks and to log a sleep diary through chatbot conversations.

### Ethical Approval

This study was approved by the Institutional Review Board (DJDSKH-17-BM-12) of Daejeon Korean Medicine Hospital of Daejeon University and written informed consent was obtained from all participants.

### PSQI Survey

The Korean version of the PSQI was used to measure sleep quality [51]. The PSQI consists of 18 questions divided into 7 subfactors to subjectively evaluate sleep in the past month. The PSQI survey inquired about the time going to bed and how long it took to fall asleep. The higher the PSQI total score, the poorer the sleep condition (range 0 to 21 points).

### Fitbit Inspire 2 Recordings

A Fitbit Inspire 2 (Fitbit Inc, San Francisco, CA, USA) was used to obtain the sleep life log data. Participants were instructed to wear the Fitbit Inspire 2 for 24 hours a day for 2 weeks to measure the amount of activity and sleep efficiency during the day; the Fitbit could be worn on either the right or left wrist according to the participant’s preference. However, the participants were instructed to take off the Fitbit when in the water for a long time, such as showering and swimming. The participants were instructed to sync their Fitbit app every morning after waking up. The data stored on the Fitbit server were collected using the Fitbit web application programming interface. The sleep information provided and collected by Fitbit included sleep variables such as the time the user fell asleep, woke up, TST, times of waking up during sleep, and sleep stages (wake, rapid eye movement, light, and deep). The administrator checked the participants’ Fitbit data after 14 days, and if the Fitbit-wearing duration was less than 10 days, they were instructed to add 7 or 14 days.

### Chatbot Conversation Recordings

**Figure 1:**
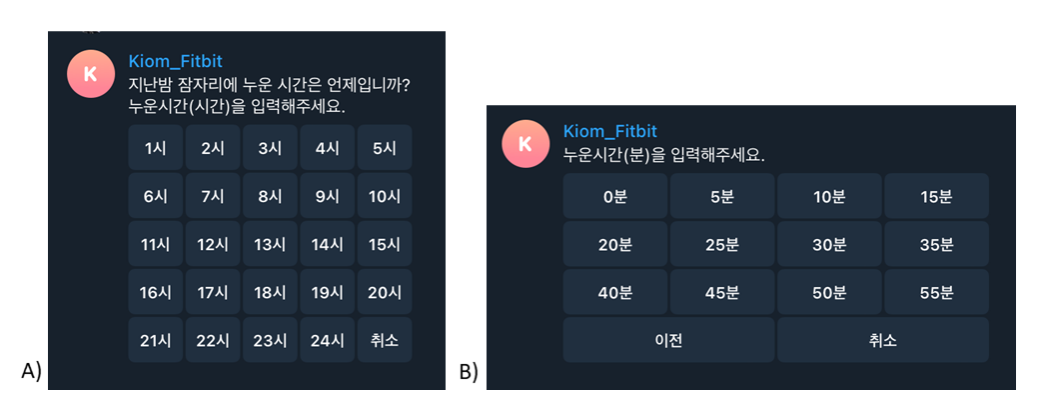
Screenshots of the Kiom_Fitbit Telegram-based chatbot app. Question-and-answer screen about the (A) hour and (B) minute of going to bed the night before.

While wearing the Fitbit, the sleep diary data of the study participants were obtained using a Telegram-based chatbot. The participants installed Telegram, added and registered a chatbot channel, and were requested to conduct conversations for 2 weeks. The participants received questions from the chatbot at 9 AM and logged sleep diaries by responding to these questions. Through chatbot conversations, the participants were asked about the time they went to bed, when they fell asleep, when they awoke, how many times they woke up during sleep, how long they actually slept, the quality of the sleep, whether there was any strenuous physical activity during the day, and how long they spent sitting. Opportunities for correction were provided, with the function of returning to the previous step during the answer and reviewing the content of the answer after the end of the conversation. The chatbot was implemented using the Python-Telegram-Bot (version 20.3) [52] (Figure 1).

For answers related to time, hours and minutes were divided and entered by clicking a button. In the case of hours, 24 buttons were presented from “1:00” to “24:00,” and in the case of minutes, 12 buttons were presented from “0 min” to “55 min.” The participants were asked whether their quality of sleep was “Very good,” “Quite good,” “Quite poor,” and “Very poor,” whereas “Yes” and “No” buttons were presented for the presence or absence of strenuous physical activity. Regarding the number of awakenings, 26 buttons were presented ranging from “1” to “more than 26.” When participants were asked to wear the Fitbit, they were instructed to continue the chatbot conversations for the same period.

### Statistical Analysis

The *startTime*, *endTime*, *endTime–startTime*, and *minutesAwake* values were used as the variables representing sleep onset, sleep offset, TST, and WASO from the Fitbit data [53]. The time of falling asleep, waking up, and actual sleep time (AST) in the chatbot response were compared to the Fitbit data. The calculated sleep time, obtained by subtracting the time of falling asleep from the time of waking up, was used as the TST, and the AST in the chatbot response was compared with the time obtained by subtracting the Fitbit WASO from the Fitbit TST.

For each participant, only the sleep information on the day when both the Fitbit data and chat responses were obtained was used and the mean value was used for each participant. To calculate the mean of the time values, the time information was converted into seconds; if necessary, 24 hours was added to prevent errors and later subtracted. The mean difference (SD) was used to compare the Fitbit data and chatbot responses, and box plots and Bland-Altman plots were used for visualization.

To observe the response characteristics of the participants, the values from 0 to 59 minutes were calculated in 5-minute increments from the response time values of the participants. Responses to the PSQI survey and Fitbit data were collected in close proximity in units of 5 minutes. Although the number of sleep days obtained by each participant differed, all the frequencies of the participants were cumulatively collected.

All statistical analyses and data processing were performed in Python (version 3.9) [54]. The PSQI survey results and chatbot responses were exported to Microsoft Excel files and read using the *pandas* tool library (version 1.5.3). Data imported from the Fitbit server were stored in an Oracle database, separated by a delimiter, exported as a CSV file, and read using *pandas* [55]. The *pandas* and *NumPy* packages (version 1.24.1) were used for data processing [56]. Box plots were drawn using the *matplotlib* library (version 3.6.3), Bland-Altman plots were drawn using the *statsmodels* *Python* module (version 0.13.5), and *P* values were calculated using *SciPy* (version 1.10.0) [57-59].

## Results

### Participant Selection Conditions and Demographic Characteristics

Participants were recruited for approximately 2 years, from October 10, 2020, to November 9, 2022. A total of 736 participants participated in this study and agreed to wear the Fitbit device for 2 weeks. Among them, 731 (99.3%) participants acquired the Fitbit data and collected the main sleep data defined by the Fitbit. During the first 14-day wearing request, 589 (80.0%) participants collected sleep data for 10 or more days. By requesting 1 or 2 weeks of additional wear, 63 (8.6%) participants collected sleep data for 10 or more days. As a result, 652 (88.6%) participants acquired main sleep data for 10 or more days, while 79 (10.7%) participants obtained less than 10 days of sleep data.

Of the 652 participants, 150 provided Fitbit data for 10-14 days and 502 provided data for 15 days or more. For participants whose data were collected for more than 14 days, only data up to 14 days were used for the analysis (Table 1).

**Table 1:** Number of days of Fitbit sleep data provided by the study participants (N=652).

Days of sleep data			Participants, n (%)
0 (Fitbit not worn)			5 (0.7)
**Less wear than requested**			79 (10.7)
	1-3	27 (0.4)	
	4-6	25 (0.4)	
	7-9	27 (0.4)	
**Worn as requested**			150 (20.4)
	10	13 (0.2)	
	11	13 (0.2)	
	12	20 (0.3)	
	13	36 (0.6)	
	14	68 (0.7)	
**Worn more than requested^a^**			502 (68.2)
	15	75 (11.5)	
	16-18	130 (19.9)	
	19-21	92 (14.1)	
	22	205 (31.4)	

^a^Only data for the first 14 days were included in the analysis.

**Figure 2:**
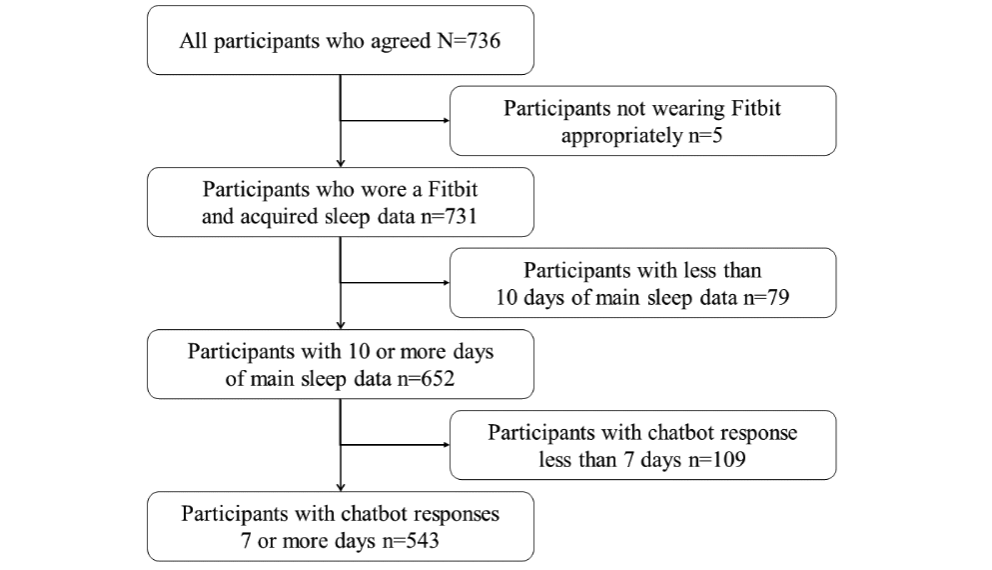
Flow of the study participants in the final analysis.

For chatbot responses, the time to go to bed, fall asleep, and wake up should be in the order of time; however, if the response value broke this order, it was considered an input error and excluded. In addition, the answerable button presented by the chatbot was set to respond to 1 of the 24 buttons from “1:00” to “24:00”; thus, responses that were considered to be wrong with respect to AM and PM were also excluded as input errors after comparison with the Fitbit data. Responses with a difference of more than 9 hours were excluded. Participants whose chatbot responses were collected for 7 or more days on the day the Fitbit main sleep data were collected were set as participants who did log chatbot responses normally. Finally, 543 (73.8%) participants’ data were analyzed, excluding 109 participants whose chatbot responses were collected over less than 7 days (Figure 2).

For the chat responses, participants responded to the sleep question sent at 9 AM in an average of 5 hours and 11 minutes, 288 (53.0%) responded within an average of 3 hours, and 505 (93.0%) responded within an average of 15 hours; 38 (7.0%) participants responded after an average of 15 hours (Table 2).

**Table 2:** Average chat response times by the study participants (N=543).

Variable	Within 3 h	Within 6 h	Within 9 h	Within 12 h	Within 15 h	After 15 h
Number of participants (%)	288 (53.0)	105 (19.3)	63 (11.6)	38 (7.0)	11 (2.0)	38 (7.0)
Cumulative number of participants (%)	288 (53.0)	393 (72.4)	456 (84.0)	494 (91.0)	505 (93.0)	543 (100.0)

Based on the PSQI total score, the 543 participants were divided into a good sleep group (5 points or less) and poor sleep group (more than 5 points). There were 318 (58.6%) participants in the good sleep group and 215 (39.6%) in the poor sleep group; this classification could not be made for 10 participants or the participants did not respond correctly to the questions. The breakdown of participants classified in each sleep group according to demographic characteristics is shown in Table 3. The majority of the participants were women; in terms of age, the greatest proportion were in their 40s, followed by 50s and 30s. According to BMI, most of the participants were in the normal group, preobese group, or obesity class I group; the BMI classification followed the Korean Society for the Study of Obesity Guidelines [60].

**Table 3:** Demographics of the analyzed participants and distribution of the Pittsburgh Sleep Quality Index sleep groups.

Characteristics		Total, n (%)	Good sleep group, n (%)	Poor sleep group, n (%)	Not classified, n (%)
All participants		543 (100.0)	318 (58.6)	215 (39.6)	10 (1.8)
**Gender**					
	Men	155 (28.5)	101 (65.2)	53 (34.2)	1 (0.6)
	Women	388 (71.5)	217 (55.9)	162 (41.8)	9 (2.3)
**Age (decade)**					
	30s	115 (21.2)	71 (61.7)	43 (37.4)	1 (0.9)
	40s	259 (47.7)	158 (61.0)	97 (37.5)	4 (1.5)
	50s	169 (31.1)	89 (52.7)	75 (44.4)	5 (3.0)
**BMI**					
	Underweight, <18.5	7 (1.3)	4 (57.1)	3 (42.9)	0 (0.0)
	Normal, 18.5-22.9	205 (37.8)	119 (58.0)	84 (41.0)	2 (1.0)
	Preobese, 23-24.9	129 (23.8)	75 (58.1)	52 (40.0)	2 (1.6)
	Obesity class I, 25.0-29.9	162 (29.8)	94 (58.0)	62 (38.3)	6 (3.7)
	Obesity class II, ≥30	40 (7.4)	26 (65.0)	14 (35.0)	0 (0.0)

### Time Response Characteristics for the PSQI Survey and Chatbot Responses

The frequency was calculated for the minute values of the time data of the PSQI survey, chatbot conversations, and Fitbit data. The time data represent the time participants went to bed, time they fell asleep, time they woke up, and AST. However, the time taken to fall asleep in the PSQI survey was calculated by adding the time taken to fall asleep to the time of going to bed, and the AST of the Fitbit was obtained by subtracting WASO from the TST. For the PSQI responses, 533 cases were analyzed once per participant and 6276 cases, representing all sleeps of the 543 participants, were analyzed for chatbot responses and Fitbit data.

**Figure 3:**
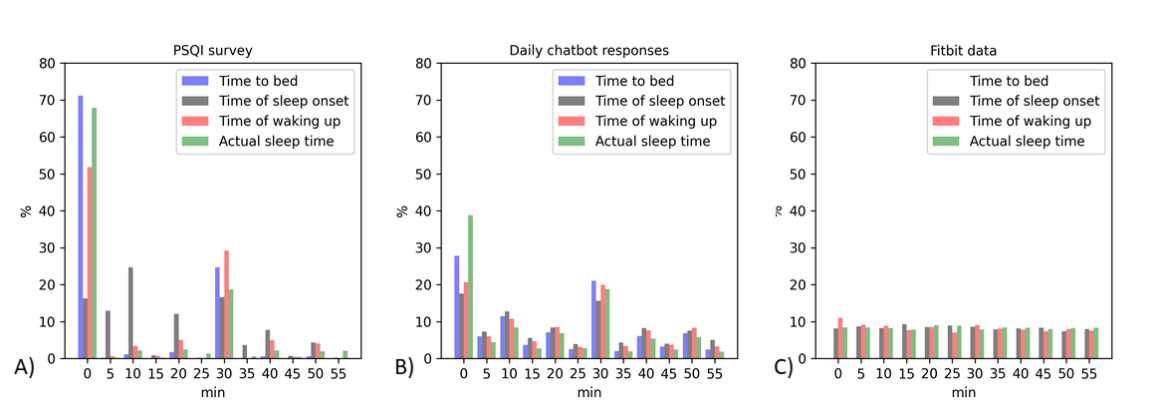
Response distribution per 5 minutes of participant data in (A) the Pittsburg Sleep Quality Index (PSQI) survey, (B) conversations with the chatbot, and (C) Fitbit data.

The proportion of respondents answering the questions related to sleep variables in the PSQI survey, chatbot, and recorded by Fitbit on the hour or in 30-minute intervals are presented in Table 4. The response distribution broken down per 5 minutes of participant data is shown in Figure 3. In the PSQI survey, most of the participants provided answers for the time they went to bed, followed by the actual sleep time and time they woke up, whereas only slightly more than one-third of participants provided the time they fell asleep. The percentages of participants responding to these questions in conversations with the chatbot were all much lower than those given on the PSQI survey, ranging from 33.2% for the time they fell asleep to 57.6% for the actual sleep time. Most respondents provided answers in 60- or 30-minute intervals; therefore, the lower response rate for falling asleep might be due to the fact that the PSQI adds the time taken to fall asleep to the time participants went to bed. The Fitbit data excluded the time when the participants went to bed; unlike the participants’ responses, similar levels of data were collected for each time period.

**Table 4:** Sleep data in 30- and 60-minute increments for Pittsburg Sleep Quality Index (PSQI) surveys, conversations with the chatbot, and acquired with the Fitbit.

Variable	PSQI survey (N=533), n (%)	Chatbot (N=6276), n (%)^a^	Fitbit (N=6276), n (%)
Time went to bed	513 (95.9)	3192 (50.9)	N/A^b^
Time fell asleep	176 (32.9)	2078 (33.1)	1045 (16.7)
Time woke up	433 (81.0)	2551 (40.6)	1254 (20.0)
Actual sleep time	463 (86.6)	3616 (57.6)	1022 (16.3)

^a^These values represent the predictable aspects of chatbot design. If the sliding interface is difficult to use, the user is also likely to leave it at 0 minutes.

^b^N/A: not applicable.

### Differences Between Chatbot Responses and Fitbit Data

**Figure 4:**
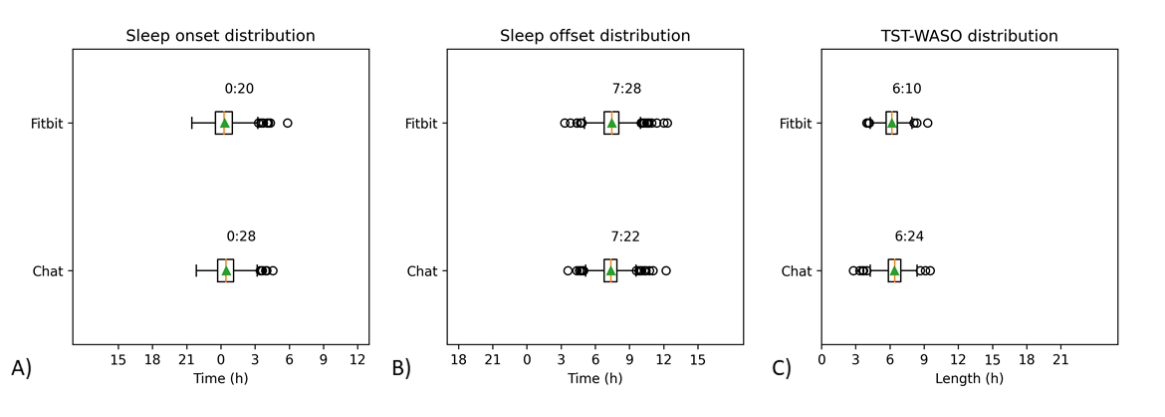
Participants’ sleep distribution obtained by chatbot conversations and Fitbit data for (A) sleep onset, (B) sleep offset, and (C) chat AST and Fitbit TST-WASO. AST: actual sleep time; TST-WASO: total sleep time-wakefulness after sleep onset.

The response distribution of participants for chatbot conversations and Fitbit data is shown in Figure 4 and Table 5. The average time the participants fell asleep by processing the chatbot conversation was 12:28:21 AM with an SD-1.96 of the difference of 65 minutes. The average time they woke up was 7:22:01 AM and the SD-1.96 was 62 minutes. The average TST calculated from the two times was 6 hours and 53 minutes (SD 49 minutes). The average time the participants fell asleep obtained by processing the Fitbit data was 12:19:42 AM (SD 70 minutes), the average time they woke up was 7:27:40 AM (SD 68 minutes), and the average TST calculated from the two times was 7 hours and 8 minutes (SD 53 minutes).

**Table 5:** Time (hour:minute:second) of falling asleep, waking up, and sleep time obtained from chatbot conversations compared to Fitbit data.

Variable	Chatbot, mean (SD)	Fitbit, mean (SD)	Difference, mean (SD 1.96)
Sleep onset	12:28:21 AM (65.1 min)	12:19:42 AM (69.7 min)	–0:08:39 (58.1)
Sleep off	7:22:01 AM (62.3 min)	7:27:40 AM (68.4 min)	0:05:38 (57.1)
TST^a^	6:53:41 AM (48.9 min)	7:07:58 AM (52.8 min)	0:14:17 (78.1)
AST^b^/TST-WASO^c^	6:23:45 (51.5 min)	6:10:06 AM (45.5 min)	–0:13:39 (87.0)

^a^TST: total sleep time (sleep off–sleep on).

^b^AST: chatbot actual sleep time.

^c^TST-WASO: Fitbit total sleep time-wakefulness after sleep onset.

The average AST answered by participants in the chatbot conversations was 6 hours and 24 minutes, which was 30 minutes shorter than the chat TST calculated by subtracting sleep onset from sleep offset and 44 minutes shorter than the Fitbit TST. Compared with the time minus WASO, the response time was 14 minutes longer. The mean difference between chatbot TST and AST was approximately 30 minutes and the average Fitbit WASO was approximately 58 minutes. The mean difference is a comparison between the Fitbit data and chat responses. In the case of sleep onset or offset, a negative value indicates a chatbot response in time later than the Fitbit and a positive value indicates a chatbot response in time earlier than the Fitbit. In the case of the chatbot TST or AST, a negative value indicated a longer time than the Fitbit and a positive value indicated a shorter time than the Fitbit (Table 5).

**Figure 5:**
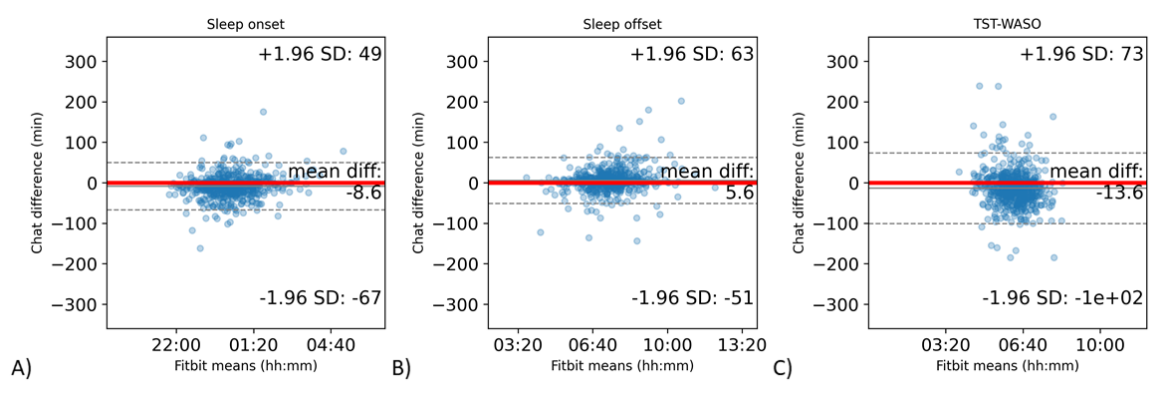
Bland-Altman plots for the time of falling asleep, waking up, and actual sleep time. The x-axis displays the Fitbit variables and the y-axis denotes the chatbot response differences based on Fitbit data. (A) Sleep onset, (B) sleep offset, and (C) chatbot AST compared to Fitbit TST-WASO. AST: actual sleep time; TST-WASO: total sleep time-wakefulness after sleep onset.

Bland-Altman statistics and plots comparing the Fitbit and chatbot responses are shown in Table 6 and Figure 5, respectively.

**Table 6:** Bland-Altman statistics for Fitbit and chatbot responses.

Variable	Fitbit–chatbot, mean	Lower LoA^a^	Upper LoA	*P* value^b^
Sleep onset	–8.6	–66.8	49.5	.04
Sleep off	5.6	–51.4	62.7	.16
TST-WASO^c^ (AST^d^)	–13.6	–100.6	73.3	<.001

^a^LoA: limit of agreement (SD 1.96).

^b^*P* values calculated from paired *t* tests.

^c^TST-WASO: Fitbit total sleep time (sleep off–sleep onset)-wakefulness after sleep onset.

^d^AST: Chatbot actual sleep time.

### Difference Between Chat AST and Fitbit TST-WASO According to the PSQI Survey and Demographic Information

According to the results of the PSQI survey analysis and the demographic classification, the mean values of chatbot TST and AST of participants by group (good sleep and poor sleep) were compared with the mean values of Fitbit TST and TST-WASO (Table 7). The results of the PSQI survey showed that the Fitbit measurement TST-WASO of the two groups was similar; however, the good sleep group responded to the AST for a relatively longer time than the poor sleep group. The AST levels in chats were similar for men and women, although the Fitbit TST or Fitbit TST-WASO was longer for women. By age, the TST-WASO measured by Fitbit was similar, although participants in their 30s and 40s indicated a longer AST than those in their 50s. There was no significant difference according to BMI, although the normal BMI group measured and responded to the AST longer, whereas the obese class I group measured and responded to the AST for relatively shorter periods. The number of participants in the underweight and obese class II groups was too small for comparison. The mean Fitbit WASO was 56 to 60 minutes (SD 21-24) in all groups.

**Table 7:** Mean differences in sleep variables determined by the chatbot and Fitbit according to sleep groups and demographic characteristics.

Variables		Chatbot				Fitbit			Difference	
		TST^a^ (h:min), mean (SD 1.96)	AST^b^ (h:min), mean (SD 1.96)	Difference (min), mean (SD)	TST (h:min), mean (1.96 SD)		TST-WASO^c^ (h:min), mean (1.96 SD)	Fitbit–chatbot TST (min), mean (SD)		TST-WASO–AST (min), mean (SD)
**PSQI^d^**										
	Good sleep (n=318)	6:59 (47)	6:34 (45)	–25 (61)	7:10 (51)		6:12 (43)	11 (68)		–21 (66)
	Poor sleep (n=215)	6:46 (52)	6:09 (58)	–37 (77)	7:04 (56)		6:0 (49)	18 (91)		–2 (108)
**Gender**										
	Men (n=155)	6:53 (44)	6:23 (45)	–30 (51)	6:56 (49)		5:58 (42)	3 (70)		–26 (68)
	Women (n=388)	6:54 (51)	6:24 (54)	–30 (75)	7:13 (54)		6:1 (46)	19 (79)		–9 (92)
**Age (decade)**										
	30s (n=115)	7:03 (49)	6:27 (49)	–35 (54)	7:07 (57)		6:11 (50)	4 (73)		–16 (71)
	40s (n=259)	6:52 (48)	6:26 (48)	–26 (71)	7:08 (52)		6:10 (45)	16 (73)		–15 (84)
	50s (n=169)	6:50 (50)	6:18 (58)	–32 (73)	7:09 (51)		6:09 (43)	19 (86)		–9 (100)
**BMI (kg/m^2^)**										
	Underweight, <18.5 (n=7)	7:14 (37)	6:39 (31)	–35 (41)	7:59 (77)		6:55 (62)	45 (159)		16 (124)
	Normal, 18.5-22.9 (n=205)	7:01 (49)	6:32 (53)	–29 (66)	7:21 (51)		6:22 (44)	20 (76)		–10 (90)
	Preobese, 23-24.9 (n=129)	6:50 (51)	6:22 (48)	–28 (80)	7:03 (50)		6:06 (42)	13 (64)		–16 (74)
	Obesity class I, 25.0-29.9 (n=162)	6:49 (47)	6:16 (54)	–33 (66)	6:58 (52)		6:00 (45)	9 (82)		–16 (93)
	Obesity class II, ≥30 (n=40)	6:45 (46)	6:15 (43)	–30 (55)	6:51 (48)		5:54 (42)	6 (81)		–22 (65)

^a^TST: total sleep time.

^b^AST: chatbot actual sleep time.

^c^TST-WASO: total sleep time-wakefulness after sleep onset.

^d^PSQI: Pittsburgh Sleep Quality Index.

**Figure 6:**
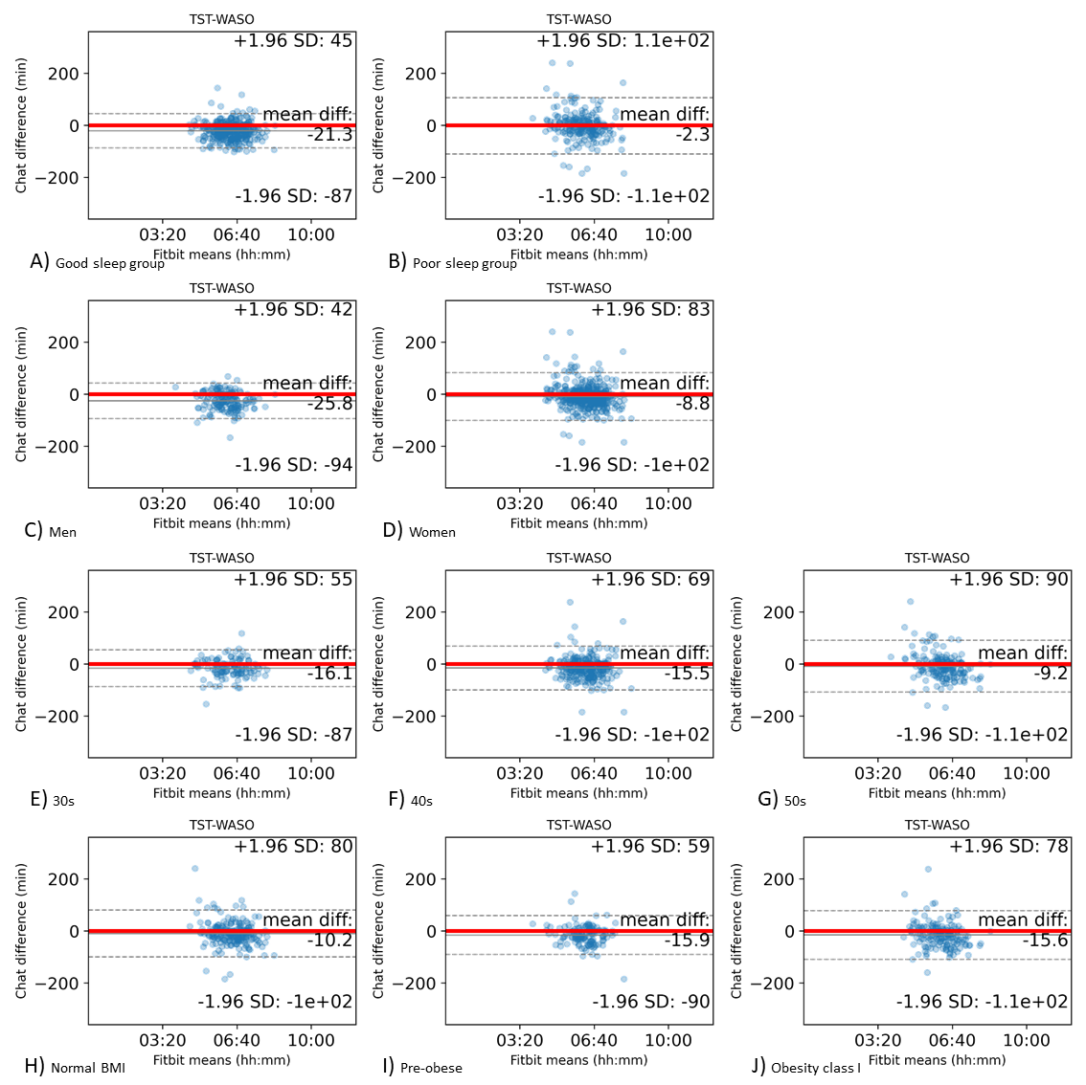
Bland-Altman plots for the Fitbit TST-WASO and the chatbot AST. The x-axis displays the Fitbit TST-WASO and the y-axis denotes the chatbot response differences based on Fitbit data. (A) Good sleep group, (B) poor sleep group; (C) men, (D) women; (E) 30s age group, (F) 40s age group, (G) 50s age group; (H) normal BMI, (I) preobese, (J) obesity class I. TST-WASO: Fitbit total sleep time-wakefulness after sleep onset; AST: chatbot actual sleep time.

The Bland-Altman statistics and plots for these comparisons are presented in Table 8 and Figure 6, respectively. The poor sleep group responded with chatbot responses that statistically matched Fitbit data, while the good sleep group reported sleeping longer than the Fitbit data.

**Table 8:** Bland-Altman statistics in sleep variables determined by the chatbot and Fitbit according to sleep groups and demographic characteristics.

Variables		TST-WASO^a^–AST^b^ (minutes), mean difference	Lower LoA^c^ (minutes)	Upper LoA (minutes)	*P* value^d^
**PSQI^e^**					
	Good sleep (n=318)	–21.4	–87.2	44.7	<.001
	Poor sleep (n=215)	–2.3	–110.5	105.9	.66
**Gender**					
	Men (n=155)	–25.8	–93.9	42.3	<.001
	Women (n=388)	–8.8	–100.6	83.0	.02
**Age (decade)**					
	30s (n=115)	–16.1	–87.0	54.8	.01
	40s (n=259)	–15.5	–99.7	68.8	<.001
	50s (n=169)	–9.2	–108.7	90.3	.10
**BMI (kg/m^2^)**					
	Underweight, <18.5 (n=7)	15.8	–107.8	139.4	.59
	Normal, 18.5-22.9 (n=205)	–10.2	–100.0	79.7	.04
	Preobese, 23-24.9 (n=129)	–15.9	–90.3	58.6	.005
	Obesity class I, 25.0-29.9 (n=162)	–15.6	–109.0	77.8	.005
	Obesity class II, ≥30 (n=40)	–21.5	–86.8	43.8	.03

^a^TST-WASO: Fitbit total sleep time-wakefulness after sleep onset.

^b^AST: chatbot actual sleep time.

^c^LoA: limit of agreement; 1.96 times the SD around the bias.

^d^*P* values calculated by paired *t* tests.

^e^PSQI: Pittsburgh Sleep Quality Index.

### Differences According to Chatbot Responses for Sleep Quality

For the chatbot responses, the days on which the participants responded “Very good” or “Quite good” for the quality of sleep the previous night were considered good sleep and the days on which they responded “Very poor” or “Quite poor” were considered poor sleep. The mean AST for good sleep responses was longer than that of the Fitbit data, and both the AST response and TST-WASO measured by the Fitbit were longer than those of the chatbot. Both the mean value of AST chatbot responses and Fitbit-measured TST-WASO of the poor sleep group were relatively short. The Fitbit WASO was 58 (SD 24) minutes for the good sleep group and was 57 (SD 35) minutes for the poor sleep group (Table 9).

**Table 9:** Mean differences between chatbot and Fitbit sleep data according to chatbot sleep quality responses.

Sleep quality	Chatbot				Fitbit			Difference	
	TST^a^ (h:min), mean (SD)	AST^b^ (h:min), mean (SD)	Difference, mean (SD 1.96)	TST (h:min), mean (SD)		TST-WASO^c^ (h:min), mean (SD)	Fitbit TST–chatbot TST, mean (SD 1.96)		TST-WASO–AST, mean (SD 1.96)
Good sleeps (n=524, 4380 nights)	7:09 (52)	6:43 (55)	–26 (88)	7:22 (58)		6:24 (50)	13 (84)		–19 (90)
Poor sleeps (n=432, 1896 nights)	6:23 (78)	5:40 (76)	–43 (121)	6:39 (88)		5:42 (75)	17 (152)		3 (151)

^a^TST: total sleep time.

^b^AST: chatbot actual sleep time.

^c^TST-WASO: total sleep time-wakefulness after sleep onset.

**Figure 7:**
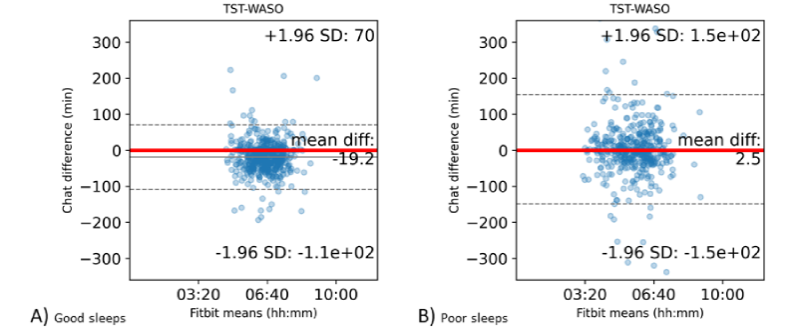
Bland-Altman plots for the Fitbit TST-WASO and the chatbot AST. The x-axis displays the Fitbit TST-WASO and the y-axis denotes the chatbot response differences based on Fitbit data. (A) good sleeps, (B) poor sleeps. AST: chatbot actual sleep time; TST-WASO: Fitbit total sleep time-wakefulness after sleep onset.

Table 10 and Figure 7 show the Bland-Altman statistics and plots, respectively. Participants who responded with poor sleeps received chatbot responses that matched the Fitbit data, whereas those who responded with good sleeps reported sleeping longer than recorded in the Fitbit data.

**Table 10:** Bland-Altman statistics comparing sleep time according to sleep quality responses in the chatbot.

Sleep quality	Fitbit TST-WASO^a^–chatbot AST^b^ (minutes)	Lower LoA^c^ (minutes)	Upper LoA (minutes)	*P* value^d^
Good sleeps (n=524, 4380 nights)	–19.2	–108.9	70.5	<.001
Poor sleeps (n=432, 1896 nights)	2.5	–148.8	153.9	.63

^a^TST-WASO: Fitbit total sleep time-wakefulness after sleep onset.

^b^AST: chatbot actual sleep time.

^c^LoA: limit of agreement; 1.96 SD around the bias.

^d^*P* values are based on paired *t* tests.

### Comparison of Fitbit TST and TST-WASO to the Participants’ Responses

To determine the probability of representing user responses using Fitbit data, we counted the number of sleeps in which the difference between the chatbot and Fitbit TST values was less than 30 minutes. In only 3483 (55.5%) of 6276 sleeps, the participants’ chat and Fitbit TST values were within 30 minutes, and only 59.0% was covered even when modified with the mean difference and quality of sleep information revealed in the previous statistics. For chat AST and Fitbit TST-WASO, less sleep was within 30 min. Even if we expanded the period to less than 60 minutes, only approximately 74% of sleeps were applicable (Table 11).

**Table 11:** Comparison of Fitbit and chatbot sleep times.

Variable	Chat TST^a^ and Fitbit TST				Chat AST^b^ and Fitbit TST-WASO^c^		
Used variables	None	Mean difference	Mean difference by QoS^d^	None		Mean difference	Mean difference by QoS
Number of sleeps within ±30 min (%)	3483 (55.5)	3,701 (59.0)	3681 (58.7)	2960 (47.2)		3174 (50.6)	3180 (50.7)
Number of sleeps within ±60 min (%)	4711 (75.1)	4816 (76.7)	4808 (76.6)	4514 (71.9)		4658 (74.2)	4661 (74.3)

^a^TST: total sleep time.

^b^AST: chatbot actual sleep time.

^c^WASO: wakefulness after sleep onset.

^d^QoS: quality of sleep.

## Discussion

### Principal Results

Whether the Fitbit was worn well for the requested 14 days was based on whether sleep data were collected for more than 10 days. Of the 736 participants who wished to participate in the study, 589 (80.0%) provided their data when first requested and 63 (8.6%) provided their data in response to the second request. The data of 543 (73.8%) participants were analyzed by limiting the number of participants who responded to chats and wore the Fitbit together for more than 7 days. In the case of chatbot responses, on average, participants responded within 5 hours and 11 minutes after the chat was delivered, with 53.0% of the 543 participants responding within 3 hours and a cumulative 91.0% responding within 12 hours.

In the Fitbit data, the distribution of data in 5-minute increments was uniform, but in the case of responses to the PSQI survey or chatbot conversations, participants had a high tendency to respond on the hour and in 30-minute increments.

The mean difference between the participants’ responses on sleep onset and sleep offset and their Fitbit data was within 10 minutes, indicating that each participant responded earlier or later within the range of up to 60 minutes compared to the Fitbit data. The mean difference in the TST was approximately 14 minutes longer for the Fitbit data. Considering that Fitbit calculates the TST by 9 minutes more and SOL by 4 minutes more, the TST of the participants, Fitbit data, and sleep diary collected through chatbot conversations were found to be quite consistent on average. However, there was a deviation of up to 80 minutes depending on the participant because of the tendency to respond in units of 30 minutes, perception of time according to the participant, and accuracy of the response accordingly.

The AST of the participants’ responses was similar to the time obtained by subtracting WASO from the TST of the Fitbit data. On average, the AST was answered 14 minutes longer than the TST-WASO of the Fitbit data and each participant showed a maximum deviation of approximately 90 minutes.

In the PSQI survey, participants were asked to describe their sleep status the month before the chatbot conversation started. When participants were divided into the good and poor sleep groups, the sleep time measured by the Fitbit was similar, although the AST of the good sleep group was longer than that of the poor sleep group. By gender, men and women responded similarly to the AST in chatbot responses, although the sleep time measured by the Fitbit was longer in women. In addition, the TST-WASO measured by Fitbit was similar according to age group; however, the ASTs for participants in their 30s and 40s were longer than those of participants in their 50s. There was no significant difference according to BMI, although the chat AST and the Fitbit TST-WASO of the normal BMI group were longer than those of the obesity class I group.

For the chatbot responses, when we compared the sleep data answered as good sleep to those answered as poor sleep, both the AST and the TST-WASO values for the good sleep group were longer than those for the poor sleep group. The AST for responses corresponding to poor sleep was almost the same as the TST-WASO.

On average for all participants, the chat response and the Fitbit data seemed to match; however, the Fitbit data could not represent the participants’ responses due to the individual differences of the participants. When tested within 30 minutes and within 60 minutes, the probability that the participant’s response was close to the Fitbit recorded data was in the 50% and 70% range, respectively.

### Limitations

Changes in Fitbit’s algorithm were not considered in this study. A Fitbit product of the same name was used; however, possible changes to the hardware or software were not considered. In addition, we cannot guarantee that the study participants wore their Fitbits and responded to the chatbot themselves, and a confirmation process for this was not included in the analysis. Sleep determined by the Fitbit was targeted as the main sleep source, and differences in sleep due to naps or occupational characteristics were not considered. We also did not take into account whether the Fitbit was worn on the participant’s dominant wrist, which could affect accuracy.

As a result of obtaining the mean difference between the Fitbit data and chatbot responses by sequentially increasing the number of days from the 1st to the 14th, the change in the mean difference according to the period was sufficiently small after approximately 7 days. Using data from at least 7 days was considered appropriate for analysis of the mean difference and SD. Considering the fatigue from continuous Fitbit wear and repeated chatting, only data from up to 14 days were used in this analysis.

In the chatbot response, if the participants fell asleep earlier than the time they went to bed or the time they woke up was earlier than the time they went to bed or fell asleep, it was regarded as an input error and was excluded. The difference between the Fitbit-measured time and reply time was very large, at approximately 12 hours, and data that could be seen as AM and PM input errors were also excluded as errors. Therefore, it is necessary to supplement the user interface to prevent user input errors. Since we did not implement a slider-like interface that allows minute input with a single touch, we did not receive every minute input. Depending on the chatbot interface, the results may vary to some extent.

The PSQI survey was conducted on sleep status during the month before the Fitbit and chatbot conversations. Under the premise that the sleep information obtained through the PSQI survey did not change rapidly, it was expected that the sleep state, based on analysis of the PSQI survey, would be applied for the next month; thus, sleep states that could not be reflected based on this assumption were not considered.

However, previous studies have shown high test-retest reliability of the PSQI. One study found that within-class correlations ranged from 0.709 to 0.813 in a retest with 30 health care workers after 2 weeks, when reliability was considered acceptable if within-class correlations were greater than 0.70 [61]. Various studies have also demonstrated high test-retest reliability of the PSQI score after 2 days or 2 to 4 weeks [62-66].

### Conclusions

There was a greater tendency to respond in 30-minute increments in the PSQI survey asking about the status of the past month than in the chatbot conversation asking about daily status. This tendency can be large when asking about the average value of past periods, which are difficult to specify, and small when asking about daily values. In addition, because this tendency is relatively small at the time of falling asleep or waking up, it can be expected to be smaller when asking about the easy-to-remember value for each day. This tendency may be greater in questions about situations that are difficult to remember or specify, such as when you went to bed and for how long you were actually asleep.

The results did not change when only the sleep data for which the chat response time was answered within 6 and 12 hours were used to determine whether the time taken to respond to the chatbot was related to the correct answer. There was no significant difference between the previous day’s sleep information answered in the morning after waking up and sleep information answered in the afternoon or evening. To reduce the causes of large interindividual variation, it is necessary to include methods that can help the process by requiring clearer queries and more accurate answers.

When the chatbot responses were compared with the sleep-related times obtained from the study participants’ Fitbit data, the mean difference for all participants was approximately 10 minutes. Considering the response rate at 30-minute intervals in chatbot responses, it can be considered that participants’ responses, on average, represented sleep time information similar to that recorded by Fitbit. Considering the distribution of PSQI sleep quality and the demographic characteristics of the study participants, the AST subjectively assessed by the participants was relatively longer than the Fitbit TST-WASO in the group with good sleep quality than in the group with poor sleep quality.

Depending on the participant, there was a deviation of up to 60-90 minutes, and it was difficult to predict whether the individual response time was earlier or later than the Fitbit data or whether the response time was short or long. This deviation may occur because each user’s sleep characteristics and response tendencies in chatbot conversations are different. It was also difficult to predict whether these differences were related to the perception of waking time during sleep, depth of sleep, or quality of sleep. It may be meaningful to provide this information or to clarify the difference between people who sleep for short periods but feel that they had good-quality sleep and people who sleep for long periods but feel that they had poor-quality sleep. It would be essential to analyze whether individuals with deep sleep patterns tend to report shorter sleep durations and whether those with shallow sleep patterns tend to report longer sleep durations to achieve similar levels of satisfaction.

If an individual’s perceived sleep time is important, their report will still be meaningful, and if their cooperation is possible, daily diary reporting will be effective. In addition, it is expected that conversations through chatbots will be able to obtain this information efficiently. To provide a clearer conclusion on the difference in user perception, it is necessary to improve the quality of sleep or depth recognition performance of wearables and to establish appropriate methods to reduce the deviation in user responses.

## Abbreviations

AST: actual sleep time
PSG: polysomnography
PSQI: Pittsburgh Sleep Quality Index
SOL: sleep onset latency
TST: total sleep time
WASO: wakefulness after sleep onset
